# How to Detect Insight Moments in Problem Solving Experiments

**DOI:** 10.3389/fpsyg.2018.00282

**Published:** 2018-03-09

**Authors:** Ruben E. Laukkonen, Jason M. Tangen

**Affiliations:** School of Psychology, The University of Queensland, St. Lucia, QLD, Australia

**Keywords:** insight, problem solving, Aha!, Eureka, creativity, surprise

## Abstract

Arguably, it is not possible to study insight moments during problem solving without being able to accurately detect when they occur (Bowden and Jung-Beeman, 2007). Despite over a century of research on the insight moment, there is surprisingly little consensus on the best way to measure them in real-time experiments. There have also been no attempts to evaluate whether the different ways of measuring insight converge. Indeed, if it turns out that the popular measures of insight *diverge*, then this may indicate that researchers who have used one method may have been measuring a different phenomenon to those who have used another method. We compare the strengths and weaknesses of the two most commonly cited ways of measuring insight: The feelings-of-warmth measure adapted from Metcalfe and Wiebe (1987), and the self-report measure adapted from Bowden and Jung-Beeman (2007). We find little empirical agreement between the two measures, and conclude that the self-report measure of Aha! is superior both methodologically and theoretically, and provides a better representation of what is commonly regarded as insight. We go on to describe and recommend a novel *visceral* measure of insight using a dynamometer as described in Creswell et al. (2016).

## Introduction

Insight is a multifaceted construct, and is better understood as an umbrella term for more objective features such as: the suddenness and unexpectedness of a solution, a non-linearity in the problem solving process, and the phenomenology of an Aha! experience. A solution to a problem can be anywhere from purely insight (sudden and unexpected), to entirely analytic. When a problem is solved analytically, one proceeds through the problem step-by-step, while conscious of their progress toward a solution. Attempts have been made to understand insight as a *feature* of certain types of creative problems that elicit insights (e.g., Weisberg, 1996; Gilhooly and Murphy, 2005), but research shows that even so-called insight problems are often solved without insight, and can be solved through a variety of strategies (Klein and Jarosz, 2011; Fleck and Weisberg, 2013; Danek et al., 2014). We agree with Danek et al. (2014) who point out that although it is well documented that some problems are more likely to be solved by insight than others, insight problems *per se* do not exist (Bowden and Jung-Beeman, 2007, were also clear in making this distinction). Therefore, a critical challenge for insight researchers is to identify when—case by case—an individual experiences an insight moment. The most popular methods are self-report, and the feelings-of-warmth (warmth) measure developed by Metcalfe (1986) and Metcalfe and Wiebe (1987). We begin by introducing both measures and our predictions. We then test the two measures for convergent validity. In the discussion, we provide advice about the general usability and conceptual merit of each measure.

### The Warmth Measure

During verbal problem solving Metcalfe (1986) asked participants to write down a number between 0 and 10 every 10 s (15 s in experiment two), where 0 is cold (far away from the solution) and 10 is hot, or certain that they had the solution. If a problem-solver exhibits gradual increases in warmth before solving the problem, then they were ostensibly aware of their progress on the problem and therefore the solution was found gradually, or step-by-step. If the problem-solver exhibits a sudden transition from a cold state to a solution, then it appears that the problem was solved through a more sudden and unexpected insight. One year later, Metcalfe and Wiebe (1987) showed that problems that had been previously categorized as insight problems showed more sudden transitions from cold states to solution states, whereas the previously categorized multi-step problems showed gradual warmth ratings preceding the solution. This contribution has had a long-standing impact on insight research and provided some of the first objective evidences that problem solving can occur in a way that resembles the insight construct. It is rare to find research on insight that does not refer to these findings, and variations of the measure are often used (e.g., Chu, 2009; Chein et al., 2010; Cushen and Wiley, 2012; Hedne et al., 2016).

### The Self-Report Measure

Asking participants to indicate, case by case, whether a problem was solved with an insight moment (i.e., suddenly, unexpectedly, and accompanied by an Aha! experience), or analytically (i.e., gradually, strategically, and step-by-step) is the most common method in recent research. In some cases a rating scale is used (e.g., Bowden and Jung-Beeman, 2003), and in other cases a retrospective forced choice paradigm (e.g., Jung-Beeman et al., 2004). Some recent research has also measured different features of the Aha! phenomenology on separate scales, which is beginning to provide a more nuanced view of the (often variable) insight experience (Danek et al., 2014; Webb et al., 2016).

### Predictions

Clearly the ideal situation is to use both the warmth and the self-report measure, and only label insights as those that are corroborated by both (as recommended by Chu and MacGregor, 2011). However, there are reasons why this solution may not be appropriate. In particular, insights can occur—at least theoretically—even when the warmth measure indicates gradual progress on the problem, as long as that progress is not related to the content of the insight (more on this in “Discussion”). The self-report measure can also detect the emotional Aha! experience, but the warmth measure can not. If the two measures are not in agreement about whether an insight occurred, at least most of the time, then using the two measures together to identify insights is not going to be productive, since many true insights would go undetected. In further support of a likely divergence between the measures, Hedne et al. (2016) found no differences in warmth ratings between self-reported insight and non-insight solutions in the case of magic tricks. Magic tricks are a relatively new way to elicit insights (Danek et al., 2014), so we should hesitate to generalize this result to the more commonplace stimuli used in insight research—i.e., classical insight problems. If the two measures do not agree, it is also appropriate to discuss which measure is likely to capture what we regard as insight, and which measure is likely to be capturing something else. We don’t have a specific prediction about the degree of convergence, but given our discussion so far, it is quite possible that the two measures do not often agree. We stress that we are *not* comparing them empirically to find out which measure is better, only to test agreement. Arguments about the merits of each measure must be made on conceptual grounds, since there is no ground truth. We will aim to provide such a perspective in the section “Discussion.”

## Methods

### Design

The participants were eighty undergraduate students (32 males and 48 females) from The University of Queensland who participated in exchange for course credit (mean age = 20.1, *SD* = 5.1). Each participant was presented with 20 verbal insight problems. We collected the insight problems from either Schooler et al. (1993), Weisberg (1996), or online sources (see Appendix A for the list of problems used). We used Weisberg’s (1996) *a priori* ‘Taxonomy for Identifying Insight Problems,’ which ensures that the problem involves *restructuring* (a re-interpretation of the problem elements, Ohlsson, 1984), and therefore is likely to elicit an insight. We used LiveCode (an open-source programming tool) to create the experiment and presented it to participants on desktop computers. The dependent variables of interest were the self-report insight measure and the feelings-of-warmth measure of insight.

#### The Warmth Measure

We calculated differential warmth in a similar way to Metcalfe and Wiebe (1987), and Hedne et al. (2016). Differential warmth is calculated by finding the difference between the first warmth rating and the last warmth rating prior to a solution. In order to be faithful to the definition of insight as a ‘sudden solution,’ we determined that an insight had occurred when there is no perceived progress on the problem before the solution, as recommended by Kounios and Beeman (2014). Whereas Metcalfe and Wiebe’s (1987) participants provided a final warmth rating that indicated that they were certain they found the solution, our participants were instructed to provide warmth ratings only before they reached the solution, and the solution itself acted as the final rating. The benefit of using differential warmth in this way, is that only two warmth ratings are required for a problem solution to be categorized as insight or non-insight, whereas the version used by Metcalfe and Wiebe (1987) required a minimum of three. Many problems are solved faster than 30 s (three warmth ratings at 10 s intervals), which means that substantial data are lost. For example, in Metcalfe and Wiebe (1987), out of 73 subjects, only 39 provided usable data. There is no foreseeable reason why our changes would result in different outcomes than the original formulation of the warmth measure and that used in Hedne et al. (2016).

#### The Self-Report Measure

We used a self-report measure of insight as recommended by Bowden and Jung-Beeman (2007). After providing a solution to a problem, participants are asked to indicate whether they experienced an insight moment by providing a rating of 1 (no), 2 (other), or 3 (yes). The 2 (other) option is for participants who guessed, experienced neither insight nor non-insight, were unsure, or did not know the answer (see Appendix B for the instructions script).

### Procedure

The research questions described in this article were assessed as part of another experiment reported elsewhere (Laukkonen and Tangen, 2017). Each participant began by watching pre-recorded instructions, and was provided with examples of insight problems. They were told that throughout problem solving, a warmth scale would appear on the right hand side of the screen every 10 s, at which point they would need to indicate how close they felt they were to solving the problem from 1 (cold/far) to 10 (hot/close). When the warmth bar appeared, the screen was locked so that participants had to immediately make a rating before continuing on the problem. The warmth bar was presented alongside a tone and participants were told not to change their rating once they had solved the problem, and to submit their response as soon as they reached the solution. The warmth bar would no longer appear once the participant started typing their answer. Participants had 1 min to complete each problem, which was presented in the center of the screen in large font, with a text box below it for typing the answer. Once a solution was provided, they completed the self-report measure of insight, and indicated whether the problem was familiar. If the problem was familiar, it was removed from further analysis.

## Results

Out of a possible 631 correctly solved insight problems, participants provided two or more warmth ratings in 180 cases. We did not include problems that were left unsolved or solved incorrectly, because omission errors and guesses were likely to add too much noise to the analysis. Initially we found a moderate to strong positive correlation between the total number of self-reported insights (*M* = 5.28, *SD* = 2.74) and the total number of warmth insights (*M* = 4.85, *SD* = 2.7) for each participant (*r* = 0.61, *n* = 51, *p* < 0.001). This indicates that self-reported insights and sudden warmth ratings are occurring approximately at the same rate, but it does not tell us whether the *same* problems were categorized as insight. To this end, we ran another Pearson’s correlation analysis across problems case by case (i.e., at the level of the question rather than at the level of participant averages). This analysis showed no significant correlation between the two measures of insight (*r* = 0.08, *n* = 182, *p* = 0.235). To provide a more nuanced perspective on the low correlation, a contingency matrix of the data is presented in **Table 1**. The contingency matrix indicates that when a sudden solution occurred according to warmth ratings, then there was a 75% chance that an insight was also self-reported by participants (i.e., 25% above chance). On the other hand, if no sudden solution was observed according to the warmth measure, then there was a 50% chance that an insight would nevertheless be self-reported.

**Table 1:** A contingency matrix representing the four possible ways that the two measures of insight can converge or diverge.

	Self-report insight	Self-report no insight
Warmth insight	A 75.6%	B 24.4%
Warmth no insight	C 50.8%	D 49.2%

*For example, Cell A represents the proportion of warmth insights where participants also reported experiencing an insight. Cell C represents the proportion of warmth non-insights where participants still report experiencing an insight*.

## Discussion

Our results indicate that agreement between the two most popular measures of insight is low or non-existent. This finding corresponds with Hedne et al. (2016) who found that warmth ratings did not differ for self-reported insights and non-insight solutions when exposed to magic tricks. A closer look at the data using a contingency matrix indicates that the primary source of divergence occurs because gradual warmth ratings have no implication on whether or not an insight is self-reported by the participants. We now consider which measure—self-report or warmth ratings—may be the better option for detecting insight moments.

Aside from the fact that there are difficulties in analyzing and comparing warmth data (see Weisberg, 1992 for a commentary on this point), there are also theoretical limitations to using warmth ratings to measure insight. One problem is that a gradual warmth pattern does not necessarily mean that an insight *did not* occur. A participant can of course make subjective progress on a problem, and therefore provide increasing warmth ratings, but then have a sudden insight that they were using the incorrect strategy followed by a solution to the problem. If this unexpected shift occurs, then the warmth ratings appear gradual and the solution predictable, when in fact it was sudden and unpredictable. There is no *a priori* reason why an insight must occur without the feeling of progress, as long as that feeling of progress is illusory or unrelated to the content of the sudden and unexpected solution. We find strong support for this perspective in our data, where participants are just as likely to report insight moments despite gradual warmth patterns.

Insights are in essence a subjective phenomenon—feelings such as pleasure, certainty, relief, drive, and surprise, are key dimensions of the insight experience that cannot be captured by warmth ratings (Danek and Wiley, 2017). Experiencing an Aha! moment is becoming increasingly the core feature of both definitions and measures of insight among researchers in the area (Bowden and Jung-Beeman, 2007; Kounios and Beeman, 2014; Webb et al., 2016; Danek and Wiley, 2017). This also means that, in a hierarchy of measures, the self-report measure of insight will take precedence. If self-reported insights consistently contradict warmth measures, then we would be forced to conclude that the warm measure is not capturing insights. Of course, if the subjective rating of insight fails to map onto anything objective, then it may not be a useful or interesting construct. Fortunately, we now know that self-reported insights map onto different eye-movements (Salvi et al., 2015), different cognitive strategies (Kounios et al., 2008), different neural activity (Bowden and Jung-Beeman, 2003; Jung-Beeman et al. 2004, 2014; Kounios et al., 2006, 2008; Subramaniam et al., 2009), differences in accuracy (Hedne et al., 2016; Salvi et al., 2016; Webb et al., 2016), and greater positive affect (Subramaniam et al., 2009). This clear mapping onto objective measures for the self-reported insights is not matched by the warmth measure, perhaps partly because it is impractical for neural investigations (Bowden and Jung-Beeman, 2007).

One issue pertaining to self-reported Aha! moments is the way that they are described to participants prior to experiments, which may in turn impact which phenomenology the participant classifies as insight. In the literature there are notable inconsistencies, for example Cushen and Wiley (2012) focused on just two dimensions, surprise and suddenness (see also Davidson, 1995 and Bowden, 1997), whereas more recent work characterizes insight based on multiple dimensions that often include affective features such as pleasure, certainty, and relief (e.g., Jung-Beeman et al., 2004; Webb et al., 2016; Danek and Wiley, 2017). Danek and Wiley (2017) recently compared experimentally the extent to which different dimensions used in previous research predict participants global Aha! ratings, thus providing a more objective mapping of the insight phenomenology. It is likely that empirically mapping the subjective Aha! experience—as in Danek and Wiley (2017)—will eventually mitigate inconsistencies and ensure more representative descriptions of insight.

### A Visceral Alternative

According to Creswell et al. (2016), “visceral states call for visceral measures.” The authors proposed that the feeling of hunger, like many other non-verbal experiences, is difficult to put into words. It is also known that verbalization can be disruptive to both task performance and subsequent memory (e.g., Schooler and Engstler-Schooler, 1990; Schooler, 2002, 2011; Brown et al., 2014). To solve this problem, the authors tested whether handgrip pressure over time—as measured by a dynamometer—could be used as a visceral, non-verbal alternative to the commonly used self-report measures of hunger. They found that the visceral measure was a better predictor of subsequent eating behavior than the self-report scale, and was sensitive to a well established food cue exposure paradigm. We propose that the insight experience is also visceral in nature, and may therefore be better captured by a visceral measure that does not interfere with the primary task. To illustrate, a participant can be instructed to begin problem solving with their hand resting on the dynamometer without squeezing, and then be asked to increase grip strength as they make progress on the problem, where a stronger squeeze is equivalent to a higher warmth rating, and a full strength squeeze indicates that an Aha! moment occurred. If the participant solved the problem, but did not experience an Aha! moment, then they can simply release their grip, indicating that the solution was found without the insight phenomenology. With these simple instructions, the dynamometer can provide continuous ratings of progress on a problem (feelings-of-warmth), and can show clearly when an Aha! moment occurs—a light squeeze followed by the sudden onset of a full strength squeeze.

## Conclusion

We believe the feelings-of-warmth measure captures only a fraction of the insight solutions that can occur during problem solving, and since the warmth measure does not show agreement with the self-report measure, it may fail to capture some crucial features of the insight experience—namely the Aha! moment. The warmth measure remains an innovative and objective measure of progress during problem solving. We recommend that warmth ratings be used to measure perceived progress on a problem, but that concluding that an insight has or has not occurred without other converging evidence is likely premature. Given the strengths of the self-report measure described as well as the relative ease with which it is administered, it is likely that self-report will continue to be the most popular method for detecting insight moments, and justifiably so. As a promising alternative, we propose that the dynamometer as employed by Creswell et al. (2016) can achieve the best of both worlds by providing an embodied continuous measurement of progress on the problem while also capturing the sudden and ineffable moment of insight.

## Ethics Statement

This study was carried out in accordance with the recommendations of the Australian National Statement on Ethical Conduct in Human Research, with written informed consent from all subjects. All subjects gave written informed consent in accordance with the Declaration of Helsinki. The protocol was approved by the Human Research Ethics Committee, The University of Queensland, Australia.

## Author Contributions

RL contributed to design, data collection, data analysis, and write-up. JT contributed to design and write-up.

## Conflict of Interest Statement

The authors declare that the research was conducted in the absence of any commercial or financial relationships that could be construed as a potential conflict of interest.
