# Supplementary material for: How to Detect Insight Moments in Problem Solving Experiments
Source: Front Psychol. 2018 Mar 9;9:282. doi: 10.3389/fpsyg.2018.00282 (PMC5854655; doi:10.3389/fpsyg.2018.00282)

Appendix A

List of Classical Insight Problems Retrieved from Schooler et al., (1993), Weisberg (1996), or online sources.

1. A man in our town has married 20 women from the town. All of the women are still alive, and the man has never been divorced. Polygamy is illegal in our town, and yet the man has broken no law. How is this possible?
2. An unemployed woman did not have her driver's license with her. She failed to stop at a railroad crossing, then ignored a one-way traffic sign and traveled three blocks in the wrong direction down the one-way street. All this was observed by a policeman, who was on duty, yet he made no effort to arrest the woman. Why?
3. Our basketball team won 72-49, and yet not one man scored as much as a single point. How is that possible?
4. One morning a woman's earring fell into a cup that was filled with hot coffee, yet her earring did not get wet. How could this be?
5. A magician claimed to be able to throw a ping pong ball so that it would go a short distance, come to a dead stop, and then reverse itself. He also added that he would not bounce the ball against any object or tie anything to it. How could he perform this feat?
6. A young boy turned off the lights in his bedroom and managed to get into bed before the room was dark. If the bed is ten feet from the light switch and the light bulb and he used no wires, strings, or other contraptions to turn off the light, how did he do it?
7. Professor Bumble, who is getting on in years, was driving along in his old car when suddenly it shifted gears by itself. He paid no attention and kept on driving. Why wasn't he concerned?
8. Mr. Hardy was washing windows on a high-rise office building when he slipped and fell off a sixty foot ladder onto the concrete sidewalk below. Incredibly, he did not injure himself in any way. How is this possible?
9. A father and his son get in a car accident. The father is sent to one hospital, and the son is sent to another. When the doctor comes in to operate on the son, the doctor says, "I cannot operate on him. He is my son." How can that be?
10. A murderer is condemned to death. He has to choose among three rooms. The first is full of raging fires, the second is full of assassins with loaded guns, and the third is full of lions that haven't eaten in 3 years. Which room is safest for him?
11. A man pushed a car. He stopped when he reached a hotel at which point he knew he was bankrupt. Why?
12. 1988 pennies are worth more than 1983 pennies. Why?
13. Sid shady works for a large construction company that was very concerned about employee theft. Someone tipped off the company that Shady was the man to watch. Each night he passed through security with a wheelbarrow full of scrap lumber, discarded electrical wires and chunks of concrete. The security guards checked the contents daily but could find nothing of value. What was Shady stealing?
14. Bobby had not taken anything and was feeling fine but he couldn't help repeating everything Mr. Jenkins said. Why is that?
15. A man walked into a bar, and before he could say a word he was knocked unconscious. Why?
16. While on safari in the wild jungles of Africa, Professor Quantum woke one morning and felt something in the pocket of his shorts. It had a head and tail but no legs. When Quantum got up, he could feel it move inside his pocket. Quantum, however, showed little concern and went about his morning rituals. Why such a casual attitude toward the thing in his pocket?
17. A prisoner was attempting to escape from a tower. He found in his cell a rope that was half long enough to permit him to reach the ground safely. He divided the rope in half, tied the two parts together, and escaped. How could he have done this?
18. If you have black socks and brown socks in a drawer, mixed in a ratio of 4 to 5, how many socks will you have to take out to make sure that you have a pair of the same colour?
19. Two men played five full games of checkers and each won an even number of games, with no ties, draws, or forfeits. How is that possible?
20. Captain Scott was out for a walk when it started to rain. He did not have an umbrella and he wasn't wearing a hat. His clothes were soaked yet not a hair on his head got wet. How could this happen?

Appendix B

Insight Instructions Transcript

Each participant received video instructions that included text on multiple slides and voiceover. The slides describing the Aha! experience—which were also read aloud in the video—are presented below. The more affective components were avoided because the warmth measure inherently cannot capture them.

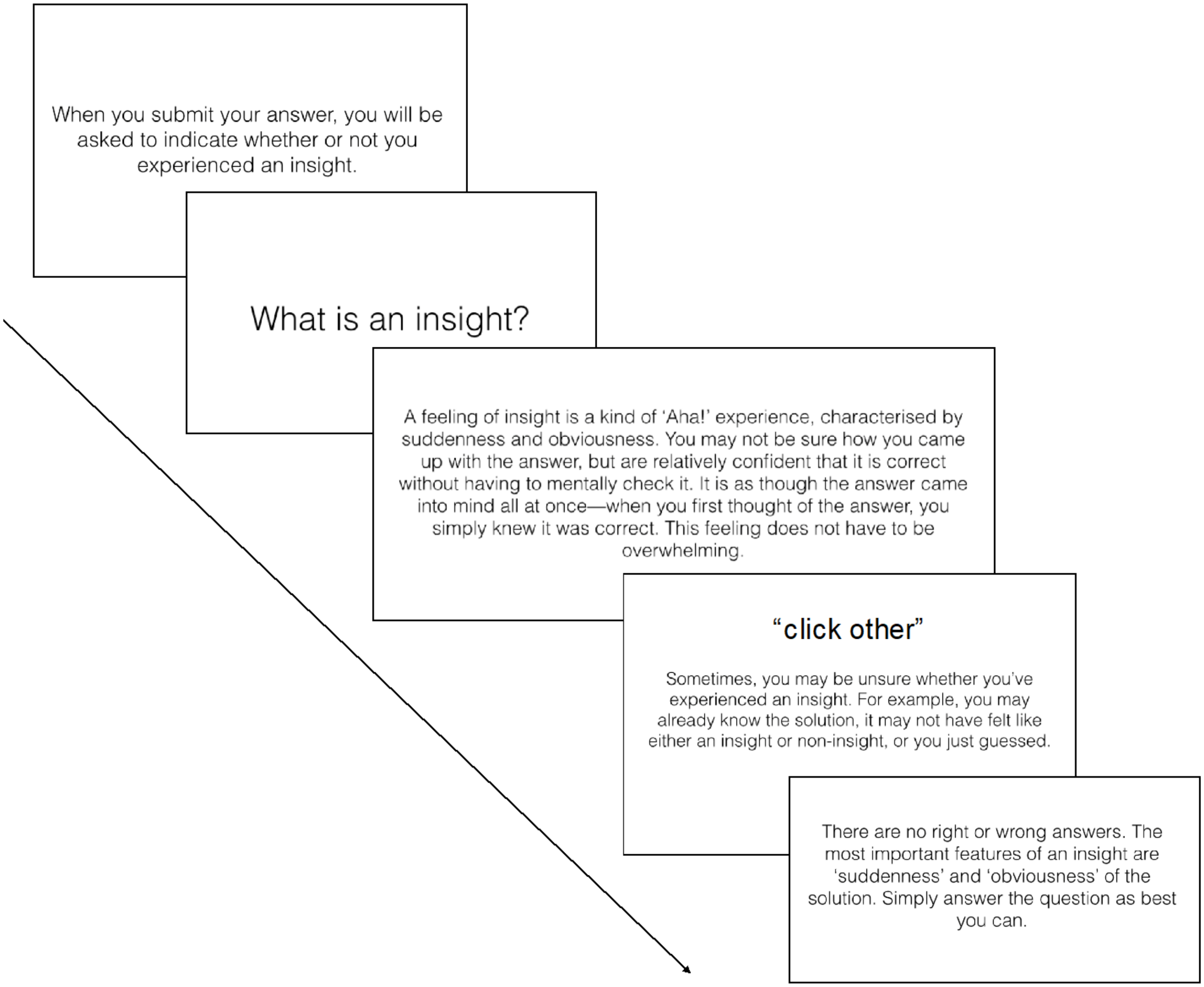

Supplement: Supplementary file 1 [file Data_Sheet_1.pdf]
